# Can We Optimize Arc Discharge and Laser Ablation for Well-Controlled Carbon Nanotube Synthesis?

**DOI:** 10.1186/s11671-016-1730-0

**Published:** 2016-11-18

**Authors:** Rasel Das, Zohreh Shahnavaz, Md. Eaqub Ali, Mohammed Moinul Islam, Sharifah Bee Abd Hamid

**Affiliations:** 1 Nanotechnology and Catalysis Research Center, University of Malaya, 50603 Kuala Lumpur, Malaysia; 2 Department of Biochemistry and Molecular Biology, University of Chittagong, 4331 Hathazari, Bangladesh

**Keywords:** Carbon nanotube synthesis, Arc discharge, Laser ablation, Process control, Optimization

## Abstract

Although many methods have been documented for carbon nanotube (CNT) synthesis, still, we notice many arguments, criticisms, and appeals for its optimization and process control. Industrial grade CNT production is urgent such that invention of novel methods and engineering principles for large-scale synthesis are needed. Here, we comprehensively review arc discharge (AD) and laser ablation (LA) methods with highlighted features for CNT production. We also display the growth mechanisms of CNT with reasonable grassroots knowledge to make the synthesis more efficient. We postulate the latest developments in engineering carbon feedstock, catalysts, and temperature cum other minor reaction parameters to optimize the CNT yield with desired diameter and chirality. The rate limiting steps of AD and LA are highlighted because of their direct role in tuning the growth process. Future roadmap towards the exploration of CNT synthesis methods is also outlined.

**Electronic supplementary material:**

The online version of this article (doi:10.1186/s11671-016-1730-0) contains supplementary material, which is available to authorized users.

## Review

### Introduction

Carbon nanotubes (CNTs) are one of the most fascinating and enchant nanomaterials of the twenty-first century [1] with many attractive physicochemical properties such as high mechanical (elasticity ~1 TPa and tensile strength 50–500 GPa), thermal stability (>700 °C), and electrical conductivity (3000–3500 W m^−1^ K^−1^) [2–4]. Since the material first defined by Iijima [5] in 1991, CNTs have demonstrated magnificent shoot in many disciplines including polymer and composites [6], conductive cable fibers and thermoplastics [7], hydrogen storage media [8], biomedical sciences [9], field emission devices [10], environmental remediation [11–13], electrochemistry and nanosensors [14, 15], nanoelectrode arrays or microarrays [16, 17], optoelectronic devices [18], catalyst supports [19, 20], and numerous others [21, 22]. Recently, National Aeronautics and Space Administration (NASA) scientists and others have explored the gold rush possibility for CNT applications in aerospace research, especially for enhanced radar adsorption. CNTs have been explored for fabricating space elevator, aircraft body, flexible car; portable X-ray machine and light-emitting diode (LED) display of various tiny devices and inspiring results have obtained [23]. The materials have a great demand in current century and are expected to be spiraling over the years.

Although CNT synthesis is considered to be a matured technology, every year, it has continued to bring hundreds of publications and patents along with new methodology. Golnabi [24] compared the total number of published papers and patents in the period of 2000–2010. During this period, an annual increase of CNT research was 8.09% for paper and 8.68% for patents in different languages and is increasing day by day. The novel and innovative applications of these materials are rapidly expanding to feed the current and future needs.

Although many methods such as arc discharge (AD) [25], laser ablation (LA) [26], chemical vapor deposition (CVD) [27], electrolytic [28], hydrothermal [29], and template [30] have been documented over the time, AD, LA, and CVD are the most widely used methods for the CNT production. However, each of them has both advantages and disadvantages. Firstly, the major benefit of AD technique is the production of a large quantity of CNT, and they have fewer structural defects than those produced by low-temperature techniques, e.g., CVD. Most of the synthesized CNTs in AD are perfectly straight as compared with the kinked-type CNTs obtained by CVD processes [31]. But the main drawback of AD is its little control effect over the CNT alignment (i.e., chirality), which is important for their characterization and application [32]. Additionally, purification of the obtained CNTs is a must, since the metallic catalyst and amorphous carbons needed to be removed for its application [33, 34]. Secondly, the main advantage of LA method is to produce relatively low metallic impurities to CNT as compared with AD, since the metallic atoms involved have a tendency to evaporate from the end of the CNT once it is closed. But the main drawback of LA is that the obtained CNT is not uniformly straight but instead does contain some branching. LA method is not economically advantageous because the procedure requires high-purity graphite rods and great requirement of laser powers and the quantity of CNT that can be synthesized per day is not as high as AD technique [32]. At last, as compared with LA, CVD is an economically practical method for large-scale and quite pure CNT production. The method is also easy to control of the reaction course. But most of the CNTs produced using CVD are more structurally defective than those produced by AD or LA methods [35].

Here, we emphasize on AD and LA, since most of the researchers often overlooked the possibilities of these two methods in order to prioritize CVD. Most of the CNTs produced by CVD are multi-walled carbon nanotubes (MWCNT), and they are riddled with defects. It is also difficult to control their chirality and diameter. Such CNTs are not suitable to fabricate industrial products, e.g., opto(electronics) and LED displays. Since industrial requirement of single-walled carbon nanotubes (SWCNT) is increasing day-by-day, a lot of puzzles, confusions, and lack of understanding of AD and LA methods should be urgently addressed for CNT yield optimization. Proper understanding of CNT growth mechanism has still remained opaque [36]. An ample literature search confirms us that huge volume of literatures includes several books and reviews describing the synthesis of CNTs through various routes [37–47]. However, we found that most of them are unnecessarily verbose and have failed to sketch a clear picture of AD and LA methods. Most of them have dealt with a single method, leaving a pitfall to the potential readers to understand the others. Here, we minimize the textual discussion and resort to tables, illustrations, and figures in order to clearly display updates and in-depth knowledge on experimental attempts of AD and LA. In the “Growth Mechanism of CNTs” section, we describe the CNT novel growth mechanisms and suggest some clues for growth control. Reactor designs and experimental evidences of AD and LA process optimizations are critically discussed in the “CNT Growth in AD” and “CNT Growth in LA” sections, respectively. The rate-limiting steps of each method are highlighted over there because of their role in tuning the growth process. The “Chiral Controlled CNT Synthesis Using AD and LA” section shows how to control the chirality of CNTs using AD and LA. Future roadmap towards the exploration of CNT synthesis methods is also outlined in the “Conclusions” section.

### Growth Mechanism of CNTs

All common CNT synthesis methods such as AD, LA, and CVD require similar catalysts to grow CNTs. Therefore, they might share a common growth mechanism. The actual growth mechanism is unclear, and it has been remained a debatable issue among the scientists. Although several mechanisms have been proposed [48–55], the detailed reaction mechanism leading to CNT formation has not been resolved clearly. Typically, two general routes, namely base-growth model [56] and tip-growth model [57], have been documented over the years. The overall process involves three main steps: (i) carbon feedstock is supplied on catalyst surface to get fullerenes as intermediate, (ii) scoot (small carbon fragments like C_2_ and C_3_) is generated from the decomposition of hydrocarbons by heat and subsequently is deposited on catalyst surface, and (iii) finally, nanotube grows until the metal is fully covered with excess carbon, its catalytic activity ceases, and the CNT growth is stopped.

Recently, Nessim [58] proposed CNT growth mode as “growth in place” and “growth then place” methods. In growth in place mode, the nanotube is synthesized on catalyst–substrate interfaces. CVD is commonly used for it. The advantages of growth in place mode include (i) control and tuning of CNT position on catalyst dots, (ii) opportunities for vertically aligned CNTs (VACNT), (iii) good physical interactions with substrate, and (iv) rapid growth. On the other hand, in “growth then place mode” CNTs are prepared through AD and LA (substrate-free CNT synthesis methods). The synthesized tubes are then separated from each other as SWCNTs or few-walled carbon nanotubes (FWCNT) or MWCNT, and the individual nanotube is purified and transferred to a pre-defined area of target substrate. Various techniques have been found to be effective for the deposition of CNTs on substrate such as dielectrophoresis [59], lithographic [60], and alternating electric fields. The main advantages of growth then place mode are (i) simple process, (ii) no/low-temperature requirement, and (iii) selectivity, purity, fabrication, and ease of functionalization. The major disadvantages of the method include compromised robustness and complicated transfer mechanism of CNTs to specific regions of substrate.

To improve further understanding of CNT growth, different researchers have proposed different routes through which CNT can be synthesized. A clear illustration for a quick and comprehensive understanding of various CNT growth mechanisms is shown in Additional file 1: Figure S1. It displays many routes such as (i) screw-dislocation-like (SDL) model [61]; (ii) weaving a rug model [62]; (iii) growing CNT as metal particle deformation and C-metal interface [63]; (iv) MWCNT nucleation and growth [63]; (v) SWCNT nucleation and growth [63]; (vi) carbide phase of SWCNT growth inside MWCNT as hybrid [64]; (vii) highly plausible growth scenario for the formation of SWCNT and MWCNT catalyzed by metal particles [64]; (viii) formation of hexagonal and pentagonal rings through metal–carbon interactions [65]; (ix) vapor–liquid–solid (VLS) growth mechanism of SWCNT [66]; (x) solid–liquid–solid (SLS) mechanism of SWCNT nucleation and growth [67]; (xi) nucleation mechanism of a SWCNT from a metal cluster [66]; (xii) effect of carbon insertion rate on the growth process [66]; (xiii) cyclodehydrogenation of the SWCNT end-cap precursor molecules and the subsequent growth of the CNT [68]; (xiv) mode of carbon diffusion [69]; (xv) hill, nanocavity, and shell of thickness of root growth model [70]; (xvi) mode of actions of SWCNT growth on a metal catalyst [71]; (xvii) SWCNT growth and chirality selection induced by single C atom and C_2_ dimer addition under catalyst-free conditions [72]; (xviii) vapor–solid–solid (VSS) [73]; (xix) cycloparaphenylenes as templates for rapid CNT formation [74, 75]; (xx) diffusion of coming carbon species on nanoparticles [76]; (xxi) growth mechanism of the aligned carbon nanotubes [77]; and (xxii) wire-to-tube model in catalyst-free CVD method [78].

Recently, Mohammad [70] attempted nine grassroots rules governing CNT growth mechanisms. The author used theoretical models with experimental evidences for exploring, especially, the VACNT of narrow chirality distributions. In summary, it was shown that the high-energy sites (HET) of foreign element catalytic agent (FECA) and substrate nanoparticle (SUBSANO) determined the catalytic decomposition of carbon source precursors (Rs) which should be unstable under the influence of HETs (rule 1). The nanoparticles (catalyst and substrate) regulate the diffusion of coming Rs species through two pathways such as bulk and surface diffusions (rule 2) (Additional file 1: Figure S1 (XX)). CNT shell that is created by the diffusion of Rs species to the peripheral surface is followed by bulk diffusion of the R_S_ species through the droplet. The shell dominated in further Rs landing on nanoparticles (rule 3). Nanoparticles with high surface energy than Rs species were necessary for diffusing low surface energy Rs species to high nanoparticle’s peripheral surface energy. It passivates the surface dangling bonds and ultimately stimulates further nanotube’s growth (rule 4). The Rs species diffusion on nanoparticles is controlled by the shell morphology such as porosity. Homogenous pores with smooth diffusion could be obtained by oxygenation. It stimulates atom-by-atom assembly for small carbon fragments for CNT nucleation (rule 5). In addition to porosity, pre-saturated soluble Rs species uniform in the nanoparticle shell was important for better nanotube growth (rule 6). The solubility percentage was highest for Fe, Ni, and Co than for Au, Pd, and Re shells. The surface atoms of nanoparticles and interatomic interactions between nanoparticles and bulk atoms would make a stressful environment on their surfaces [79]. This surface stress energy mediates nanotube growth with narrow chiral distribution. The shell should have uniform distribution stress at surface which segregates R_S_ species to the nanoparticle peripheral spaces (rule 7). Rule 8 defined the feet of shell dimensions on the rate and types of CNT growth. Nanoparticles with small shell width showed the highest solubility of the R_S_ species in nanoparticle shell. Smaller nanotube wall thickness with identical width of the shell allowed fast growth rate in a trend of SWCNT > double-walled CNT (DWCNT) > FWCNT > MWCNT. Finally, CNT growth rate (GN) on nanoparticle surface decreases with increase in inverse temperature T [e.g., log (GN)/1/T^1^] (rule 9). It is because of the controlling behavior of temperature on R_S_ species permeability in the shell [76].

However, we believe a single mechanism cannot suffice the growth of all nanotube with different diameter, length, and chirality. Ways for atomic scale tuning should yet to be worked out. The tuning process might be associated with catalyst shape, chemistry, morphology, texture, and some other factors. Challenges are yet to be resolved for adding small carbon fragments called scoot at nanotube tips. Some articles reported that the addition of monomer and dimer carbon fragments to growing nanotube tips aids in the growth process. However, to the best of our knowledge, the precise nature of such a mechanism has yet to be established. The growth of hybrid or mixture of CNT has long been an encountered problem. The separation of pure CNT, especially SWCNT, from their mixtures is a challenging jobs, since they posses many common features with the MWCNTs, making the traditional separation techniques inefficient. If we can control the addition of single carbon atom to nanotube waist, it would be possible to change their properties necessary for bulk applications. For instance, we can control their electronic properties by changing their chiral angle which could be converted metallic nanotubes into semiconducting features, which we highlight in the “Chiral Controlled CNT Synthesis Using AD and LA” section. Peer view should be given to control diameter and chiral angle, since these parameters determine the metallic, semiconducting, or bandgap properties of the synthesized CNT. On the other hand, numbers of wall formation followed by their stacking force could crave the MWCNT. We believe that the complete understanding of catalyst role in nanotube growth would unravel both phenomena and would offer a programmable robust route for the synthesis of structurally uniform CNT, which are often important for specialized applications.

An ample literature study shows some research gaps, controversial hypotheses, and challenges which might be considered for understanding the better growth mechanisms and should be fixed to increase CNT yield with desired properties. Firstly, the dispute understanding of metal nanoparticle catalyst during the CNT growth are whether they remain as solid or liquid/melt state, whether the carbon diffusion in metal is volume diffusion or surface diffusion, whether the actual catalyst for CNT growth is the pure metal or metal carbide, etc. [80]. Gavillet et al. [81] proposed a mechanism for the base growth of CNT taking into account the volume diffusion of the carbon species through the metal bulk at a temperature (T) lower than the eutectic temperature (T_E_) of the catalyst eutectic alloy during growth. Most of the CNT grew in the past where T < T_E_ have raised a question how could the volume diffusion take place at T < T_E_? Could there at all be a eutectic alloy formed for this at T > TE—in violation of the rules for the formation of eutectic alloy? [76] The existing growth mechanisms could never be explained this scenario and has remained opaque. Secondly, there is no explanation previously published to broad our knowledge where and how the active R_s_ species are created from their precursor/source and nucleated to form the wall of CNT on catalyst surface. Using atomic scale in situ transmission electron microscopy (TEM) [82] during the reaction process may help in observing and understanding the nucleation process certainly. Thirdly, it is well known that carbon diffusion will take place following base growth or root growth mechanism once the CNT precipitation fails to push the metal particle up (especially in the case where catalyst nanoparticle attached strongly with the substrate). Since there is a cap on the surface of nanoparticle which has been produced at an early stage of the reaction, the cap would block the carbon species from entering the nanoparticle surface for further nucleation. Therefore, Gohier et al. [83] claimed that both the base growth and the tip growth can take place on the same nanoparticle formed on the same substrate. It suggests that the substrate-nanoparticle adhesion is not probably the real cause of base growth of CNT. Finally, there is a lack of study published on disclosing the contribution of different sizes and shapes of nanoparticle catalyst for nanotube growth. Since the metal catalyst could be grain, island, pocket, or patch due to temperature effect, they might have different shapes and size. Matsubara and Kamiya [84] observed that the vibration of atoms in a nanoparticle is more anisotropic and much larger at the top surface than at the interior of it. At high temperature, the nanoparticle surface goes through several stages of disorder. The contribution of these disorders in the sublattice might have positive and/or negative effects on carbon diffusion onto the disordered catalyst surface. Therefore, a catalyst can be crystalline, semicrystalline, and non-crystalline depending of growth temperature. Based on these hypotheses, future studies can be undertaken to tune the nanoparticle shapes, which might determine the overall chirality and diameters of CNTs created in AD, LA, and CVD methods.

### CNT Growth in AD

**Fig. 1:**
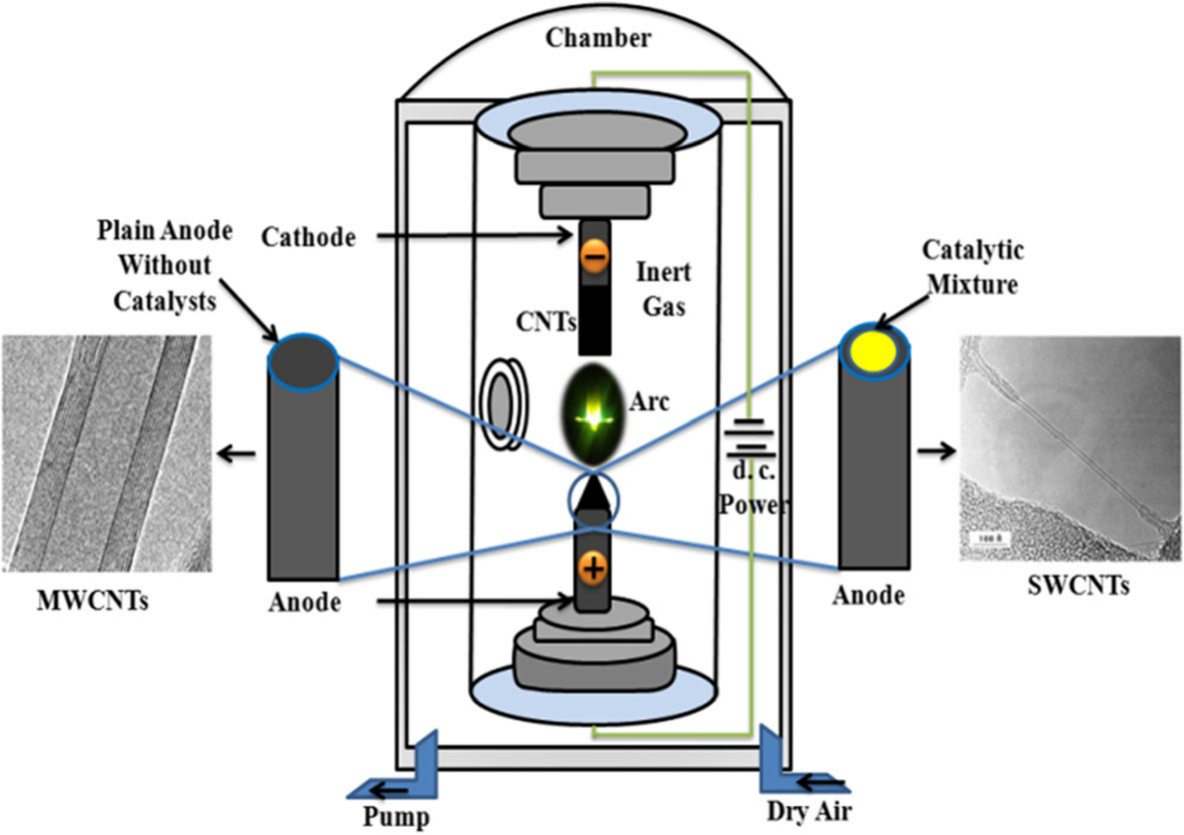
Schematic of AD apparatus for synthesizing SWCNT and MWCNT

AD is a common and simple method to synthesize CNTs, and it was initially practiced for the synthesis of fullerenes. The method synthesizes CNTs [85–88] with mixture of components like carbonaceous particles and metal catalysts, and hence, product purification is a must [89]. The principle of AD method is schematically depicted in Fig. 1. In this method, a high current (50–100 A) is applied through two graphite electrodes—anode and cathode in a closed chamber. Plasma of inert gas is ignited at high temperature (>1700 °C) and low pressure (50–700 mbar) into the chamber. The two graphite electrodes having a mean diameter of about 6–12 mm are placed face to face with a gap of 1–4 mm [90, 91]. The applied current produces small carbon fragments by disrupting graphitic carbon networks in positive anode electrode. The fragments are then simultaneously deposited on the negative cathode electrode. Thus, the length of negative anode is decreased with the initiation of CNT production on the positive cathode electrode [92]. Thus, the length and compositions of anode are directly proportional to the formation of CNTs on cathode electrode. Carbon atoms are vaporized due to high temperature and pressure and released from anode graphite rod in the form of small carbon cluster (C_3_/C_2_) [90]. Subsequently, this cluster is deposited on a precursor or metal surface catalyst on cathode electrode surface and rearranged them into microtubule-like CNT structure. However, the formation of desire CNTs (either SWCNTs or MWCNTs) largely depends on the inert gas type, temperature, current, and catalysts used in the discharge chamber, which we highlight at the end of the “Engineering Principles for CNT Yield Optimization” section.

#### Engineering Principles for CNT Yield Optimization

The synthesis of pure SWCNTs is highly desired since they have wide spread applications in electronics and biomedical fields [93, 94]. For synthesizing this special tube in AD method, anode is soaked with various metal catalysts which are not required for the synthesis of MWCNTs (Fig. 1). The anode composite determines the basic morphology of SWCNTs, since it contains either single metal (Fe, Co, Al, Ag, Mn, Mg, Pt, Pd, Ni, and so on) or metal composite (Mg–Ni, Fe–Co, Ni–Ti, Co–Au, etc.) [95, 96].

Iijima and Ichihashi [86] and Bethune et al. [87] were the first to synthesize SWCNTs using AD method. Saito et al. [97] used Ru, Pd, Rh, Os, Ir, and Pt catalysts, and Pd, Rh, and Pt demonstrated good catalytic activity with a yield of better quality SWCNTs. Thus, both the growth and quality of synthesized CNTs depend on particular metal catalyst used in the reaction vessel. In addition to metal catalysts, metal oxide catalysts (Y_2_O, C_e_O_2_, and La_2_O_3_) are also widely been practiced in SWCNT synthesis [98]. Zhao and coworkers (2010) developed an efficient and cost-effective method using charcoal as major carbon source and FeS as catalysts. SWCNTs of diameter 1.2 nm were abundantly synthesized using this charcoal substrate. The method brought down the starting material costs by approximately tenfold [99]. This group also used bituminous charcoal substrate and Ni–Y catalyst mixtures and obtained moderate yields of SWCNTs of diameter 1.2–1.7 nm [100]. CNTs were observed even in the absence of metal catalysts where a pyrite-containing coal was used as substrate. Probably, the contaminated metal, originally present in the pyrite coal, might act as a complement of the extraneous catalyst. Moothi et al. [47] extensively reviewed the coal-based CNT synthesis using AD, thermal, and plasma CVD deposition techniques.

Another cost-effective technique has recently been introduced by Xu et al. [101]. This group synthesized SWCNTs, double-walled CNTs (DWCNTs), and triple-walled CNTs (TWCNTs) from asphalt substrate—an abundant carbon source found in nature. In another experiment, Xu et al. [102] used petroleum coke-derived electrode as anodic carbon source, which was mixed with Fe powder (1:2) for synthesizing both SWCNTs (1.0–1.6 nm in diameters) and DWCNTs (3.0–4.4 nm in diameter) under N_2_, He, and Ar gas pressure 0.04–0.05 MPa. Converting petroleum coke into high value-added SWCNTs showed the unique opportunity for utilizing other cheap carbon sources as major carbon feedstocks which could decrease the overall production cost of both SWCNTs and DWCNTs in commercial premises.

**Fig. 2:**
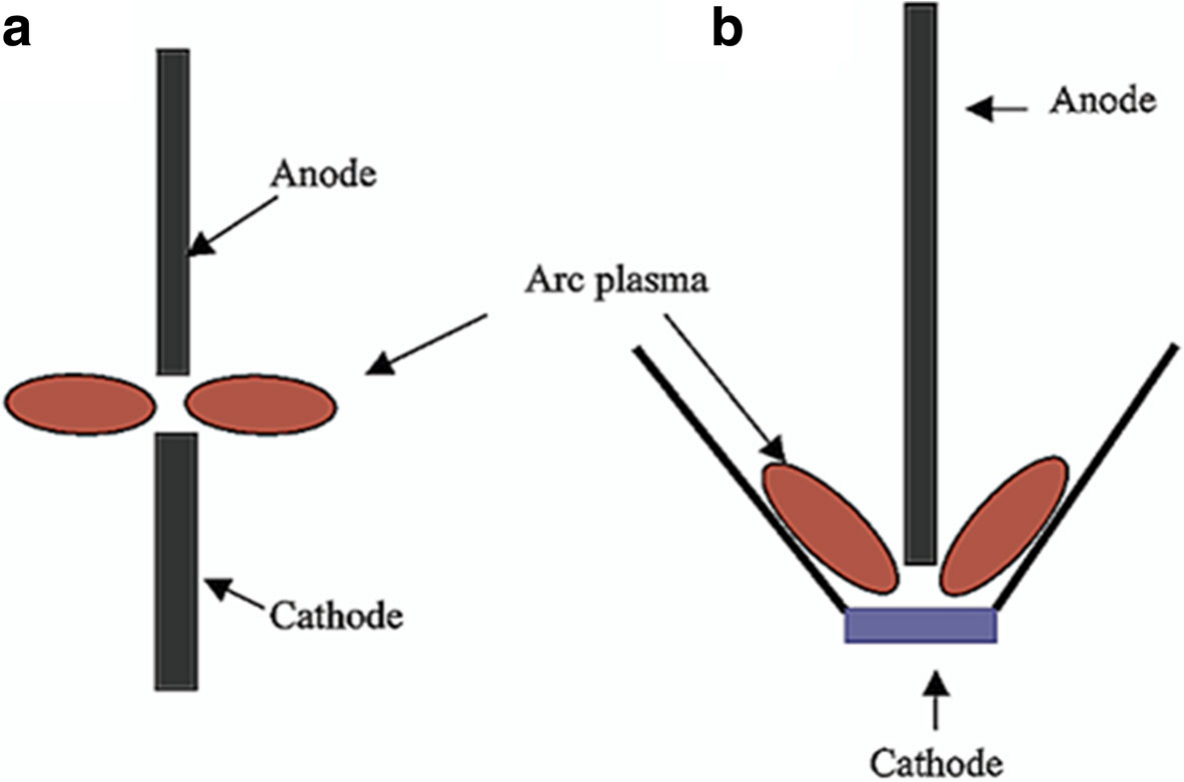
Schematic drawings of the electrode setup for **a** a conventional and **b** new bowl-based AD electrodes. The figure is adapted with permission from American Chemical Society [105]

The diameter-controlled synthesis of SWCNTs which have superior physicochemical properties is often a challenging job. Temperature plays critical role in this venture, since it causes condensation of metals and carbon atoms between surrounding plasma and cathode electrode and thus controls nanotube diameter in the reaction vessel. Using argon with lower thermal conductivity, Farhat et al. [103] obtained SWCNTs with a smaller diameter (1.2 nm). The diameter was decreased by 0.2 nm for every 10% increased in argon helium ratio when “C:Ni” and “C:Y” were used at ratio 94.8:4 and 94.8:1, respectively. Therefore, a variation in temperature causes alteration of Ar:He ratio which can bring changes in nanotube morphology. Temperature changes the growth of nanotube as it directly affects catalysts lifetime. Another mechanism to control SWCNT morphology is to adjust the distance between anode and cathode electrodes. It controls flow and enhances anode decomposition rate followed by increased SWCNT production. In CVD method, some metal catalysts such as Co, Mo, and Ni are used to synthesize low diameter (0.6–1.2) SWCNTs [98]. Application of such catalysts in AD method also produces SWCNTs with similar features [98]. These diameters are significantly smaller than the normal diameter (1.2–1.4 nm) of SWCNTs [104]. In addition to diameter control, oxidant resistant SWCNTs are often a material of choice in the field of optoelectronics. Huang et al. [105] significantly improved the AD method using a bowl-like cathode to synthesize high oxidation resistant SWCNTs with lower level of defects (Fig. 2). Magnetic field can also be applied for controlling SWCNT diameter in AD chamber [106, 107]. Yokomichi et al. [108] applied a high magnetic field (10 T) for controlling SWCNTs diameter (1.3 nm with and 0.8 nm without the magnetic fields) and increased the deposition rate.

The carbon AD method produces impure SWCNTs mixed with carbonaceous particles like fullerene, MWCNTs, FWCNTs, graphitic polyhedrons, impure ash, metal catalysts, and amorphous carbons [109]. Several methods can be applied to eliminate impurities such as wet oxidation, acid and thermal treatments, annealing, filtration, ultrasonication, and gas purification to get high-crystalline SWCNTs [46]. Jeong et al. [110] synthesized SWCNTs by AD method and purified them by thermal and acidic treatments. Highly crystalline SWCNTs with superior performances were observed upon extensive purification. Thus, the synthesis of pure, defect-free, and highly crystalline SWCNTs have been still remaining a technological challenge.

The synthesis of few-walled and double-walled carbon nanotubes (F/DWCNTs) is similar but more complicated than that of SWCNT [111]. The process much more depends on growth factors mixed in the reaction vessel. Several successful projects for the synthesis of F/DWCNTs are outlined in LA section. It is interesting to note that DWCNTs with high purity and yield (4 g/h) can also be synthesized from MWCNTs instead of graphite powder [112].

Probably, the carbon AD method is the easiest route to get MWCNTs, provided proper growth conditions are maintained. The needle-like MWCNTs were first synthesized by Iijima [5] using an AD evaporation method with DC current in argon-filled vessel under 100 Torr pressure. The size of the tube was 4–30 nm in diameter and 1 mm in length. However, pure MWCNT with high yield is difficult to obtain using AD because of the effects of different parameters such as atmosphere, current, and electrode composition; plasma and pressure are used in the reaction chamber. Different reaction factors may change the morphology of final CNT products [113]. Extensive experimental details of MWCNT synthesis following AD method was provided by Shimotani et al. [114] under four different atmospheres (He, CH_3_CH_2_OH, CH_3_COCH_3_, and C_6_H_6_) at a pressure of 50–500 Torr. The CH_3_CH_2_OH, CH_3_COCH_3_, and C_6_H_6_ atmospheres increase MWCNT production of twofold than of He atmosphere. Probably, the decomposition of CH_3_CH_2_OH, CH_3_COCH_3_, and C_6_H_6_ under high pressure and temperature contributes to free carbon and hydrogen atoms which synthesize MWCNT cluster on cathode ray tube [46]. In plasma rotating AD, the internal plasma is circulated in reaction chamber. The rotating force distributes and accelerates the vapor carbon atoms and accelerates them towards nanotube assembly. However, recent studies suggested that the effect of high pressure is more prominent than gaseous atmosphere in the synthesis of MWCNTs. Kota et al. [115] used different He pressures (80–600 Torr). The highest percentage of MWCNTs was found at 450 Torr because of its high evaporation rate in anode electrode. They also proposed a method to get highly crystalline MWCNTs at high-density graphite electrode. Such nanotube could open a new avenue in water purification fields, since CNTs can make CNT membrane which acts as hollow tube to transport water into it and simultaneously rejects multiple pollutants in water [116]. It is interesting to note that Kim et al. [117] synthesized MWCNT taps (73% crystalline, 10 nm in diameter, 2.3 μm in length) by using a movable graphite anode and a rotating highly resistive cathode in stable arcing conditions. Such tube can be used to prepare conductive plastic composite materials with ease and controlled manner. Although DC AD is popularly used as current source, pulsed techniques are also in practice. Using a 0.2-μs pulse, Parkansky et al. [118] synthesized MWCNTs with diameter of 10 nm and length up to 3 nm.

The AD between two pure graphite electrodes without any metal catalysts gives the final structure of the MWCNTs. Its morphology largely depends on homoeostatic environment of the reaction vessel. Liquid nitrogen as an atmospheric gas performs effectively [119, 120] in terms of high purity and yield. The procedure is economical since it does not demand high pressure and expensive inert gases. Rotating plasma technique is interesting useful to increase the yield [121]. It involves the uniform distribution of discharges which help to stabilize plasma in large volume. Different atmospheric gases have different diffusion capabilities that might affect nucleation of carbon clusters on cathode electrode. It creates uniform thermal conductivities, which help to grow MWCNT uniformly with different layers, diameters, and size distribution, depending on the surrounding plasma diffusion coefficient rate in the discharge reaction vessel.

In order to help the potential readers to understand the holistic approach of SWCNT, FWCNT, and MWCNT production using AD method, here, we compile some major findings as shown in Table 1. As shown in Table 1, it is clear that the uses of catalysts are a must to synthesize both SWCNT and FWCNT, whereas MWCNT can be obtained without catalyst. Three catalysts such as Fe, Ni, and Co are widely used with other catalyst promoters in the form of bi(tri)metallic nanoparticles. Although Fe nanoparticle is sufficient for SWCNT production, adding sulfur has shown important for FWCNT production. It might be due to the effects of sulfur in the development of a core/shell at the cathode which might have different melting points, and this core/shell could promote the growth of FWCNTs [122]. Helium and argon are widely used gases for SWCNT production, whereas hydrogen and argon are found popular for FWCNT production (Table 1). But we do not recommend to use pure hydrogen, since we notice it is unfavorable for mass production of S/FWCNT due to the instability of AD plasma [123]. Helium, air, and hydrogen are commonly used for MWCNT production (Table 1). Hydrogen is more suitable because it is highly thermally conductive and the uses of hydrogen could ensure highly pure CNT, since it could reduce amorphous carbon by forming hydrocarbon with them [124]. Table 1 also suggests that using helium and argon are suitable to yield high-quality and long CNT production. Average range of pressure for CNT yield shown in Table 1 is between 200 and 500 Torr but depends on the nature of gases. Pressure is required to provide energy to the gas molecules and to act as a wall for a steady flow of ions between the electrodes of AD. Helium typically requires higher pressure (500 Torr) than carbon monoxide, argon, and hydrogen. At pressure below 300 Torr, the yield is found to be very low because the density of ions would be decreased at lower pressure that might unstable AD plasma. On the other hand, at high pressure, more number of ions participates in the plasma thereby restricting the motion of carbon vapors from anode to cathode and decreases the CNT yield. As shown in Table 1, the average current density uses in AD from 50 to 200 A. The highest current density can give better yield of S/FWCNT, whereas the lowest current density can produce thick and long MWCNT. However, there are no conclusive evidences that the regulating current density can produce high quality of CNTs and thus, there is a critical need to develop a strong correlation between optimum current levels and formation of nanotubes.

**Table 1:** Reaction parameters for CNT yield optimization using AD method

Metal catalyst	Atmosphere	Pressure (Torr)	Current (A)	Major observation	Ref.
SWCNT					
Fe	CH_4_ Ar	10 and 40	200	• SWCNTs of 1 nm in diameter are obtained	[86]
Co, Fe, Ni	He	100–500	95–105	• Co helped to produce SWCNTs with uniform diameter of 1.2 nm	[87]
Ni, Pd, Pt	He	550	70	• Ni-filled anode stimulates SWCNT growth	[167]
Ni–Co, Co–Y, Ni–Y	He	660	100	• SWCNT bundle filament is secured. It consists of smaller aligned SWCNTs self-organized into bundle-like crystallites with diameters ranging from 5 to 20 nm	[88]
Y–Ni	He	100–700	40–100	• Only 40% of SWCNTs with diameter 1.3 nm are realized	[168]
Fe	H_2_–Ar	200–520	28–34 V	• Highly crystalline SWCNTs with diameter 10–30 nm are obtained	[169]
Co-Ni	He	500	80–100	• SWCNTs are synthesized with uniform diameter 1.7 nm • SWCNT production rate 7 g/h and the purity 70%	[170]
Ni, Y	Co, He	225	100	• SWCNTs with small diameter 1.66 nm are preferentially etched with the increase of Co concentration	[171]
Fe–Mo	Ar–H_2_	225	90	• SWCNTs with selected diameter distributions are secured	[172]
Fe, Co, Ni	He	300	–	• SWCNTs are obtained with a number of carbonaceous and embedded catalyst particles on surface	[110]
Fe	N_2_, He, Ar	300–375	60–80	• High-quality SWCNT, DWCNTs, and TWCNT are synthesized with different diameters • He gas supported SWCNT production • Ar gas responsible for TWCNT formation • N_2_ gas encouraged DWCNT production	[101]
Fe, W	H_2_, Ar	200	70–120	• SWCNTs are synthesized with high yield • Fe–W catalysts made SWCNT smaller than those using Fe catalyst alone	[173]
Ni/Y	Ar	12 kPa	90	• SWCNTs of diameter 1.29–1.62 nm are synthesized with higher oxidization temperature	[174]
Fe, Co, Ni, and FeS	H_2_	240	120	• De-bundled SWCNTs with diameter 3 nm are synthesized	[175]
FWCNT					
Ni, Co, F,S	Ar, H_2_	350	75–80	• DWCNTs with outer diameter (1.9–5.0 nm) and inner tube diameters (1.1–4.2 nm) are obtained	[176]
Y–Ni/Co	Ar	–	40–60	• High-quality DWCNTs with inner and outer diameters 0.8–1.2 and 1.6–2.0 nm, respectively, are realized	[177]
Ni, Co, FeS, NiS, CoS, FeS, Sn	He	600	180	• High-quality DWCNTs with diameter (2–7 nm) super bundles are selectively grown	[178]
FeS KCl	H_2_	350	70	• DWCNTs with perfect lattice structure are synthesized with high yield	[179]
Ni(HCO_2_)_2_·2H_2_O	H_2_	240	120–300	• 65% of DWCNT are obtained within 10 min with narrow diameter distribution (outer 1.98–3.47 nm and inner 1.32–2.81 nm)	[180]
Ni/Co/Fe, Ye/La	Ar	760	50	• Highly pure DWCNT (95%) are achieved	[181]
Fe, S	Air	0.75–135	90	• FWCNT are effectively synthesized with diameter 1.6–6 nm	[182]
MWCNT					
–	He	500	18	• MWCNT with diameter (5–30 nm) and length of several micrometers are secured	[85]
–	CH_4_	50	20	• Thick MWCNTs are synthesized	[113]
–	H_2_	60	50	• Fine and long MWCNTs are realized	[183]
Nd–Fe–B magnets	He, Ar, O_2_, N_2_, Air	750	~4.0 × 10^11^ A/m^2^	• Obtained MWCNTs are highly pure (>95%) • MWCNT purities are ~97% in (air), ~96% (Ar), ~40% (He), ~33% (N_2_), and ~26% (O_2_) gases	[184]
–	Liquid N_2_	–	80	• 70% of MWCNT with diameter (20–50 nm) and a few μm in length is obtained	[119]
C_8_H_10_ C_10_H_10_F	–	500	10–70	• Both (SWCNTs and MWCNTs) are obtained	[185]
Co, S, Pt	H_2_	300	100	• Environmental temperature showed a significant effect on the formation of MWCNTs as well as the diameter of the tubes	[186]
–	He Air	500	150	• Highly graphitic MWCNT (yield 60%) in He atmp. and traces of DWCNT are secured	[144]
Nd-Fe-B magnet, Co metal	Water	–	50	• Purity and quality of obtained MWCNT are both improved markedly • Co helped to get a cylinder-like CNT structure	[187]
Hydrocarbon compounds as precursors	He	300–600	30–90	• Thick MWCNTs are obtained • Aromatic hydrocarbons, including pyrene and xylene, are suggested to not only act as precursors but also enhance the growth rate of MWCNT	[188]
–	Air	60	80	• Fine and long MWCNTs are obtained free from carbon nanoparticle and graphite platelet	[189]

The quality of CNT and its rate of production in AD depend on several factors, such as supply of power, carbon precursors, atmospheric gases, catalyst types, gas pressure, and finally temperature. Here, we highlight one-by-one in the subsequent paragraphs, so that the potential readers can optimize desired CNT yield using AD method.

**Fig. 3:**
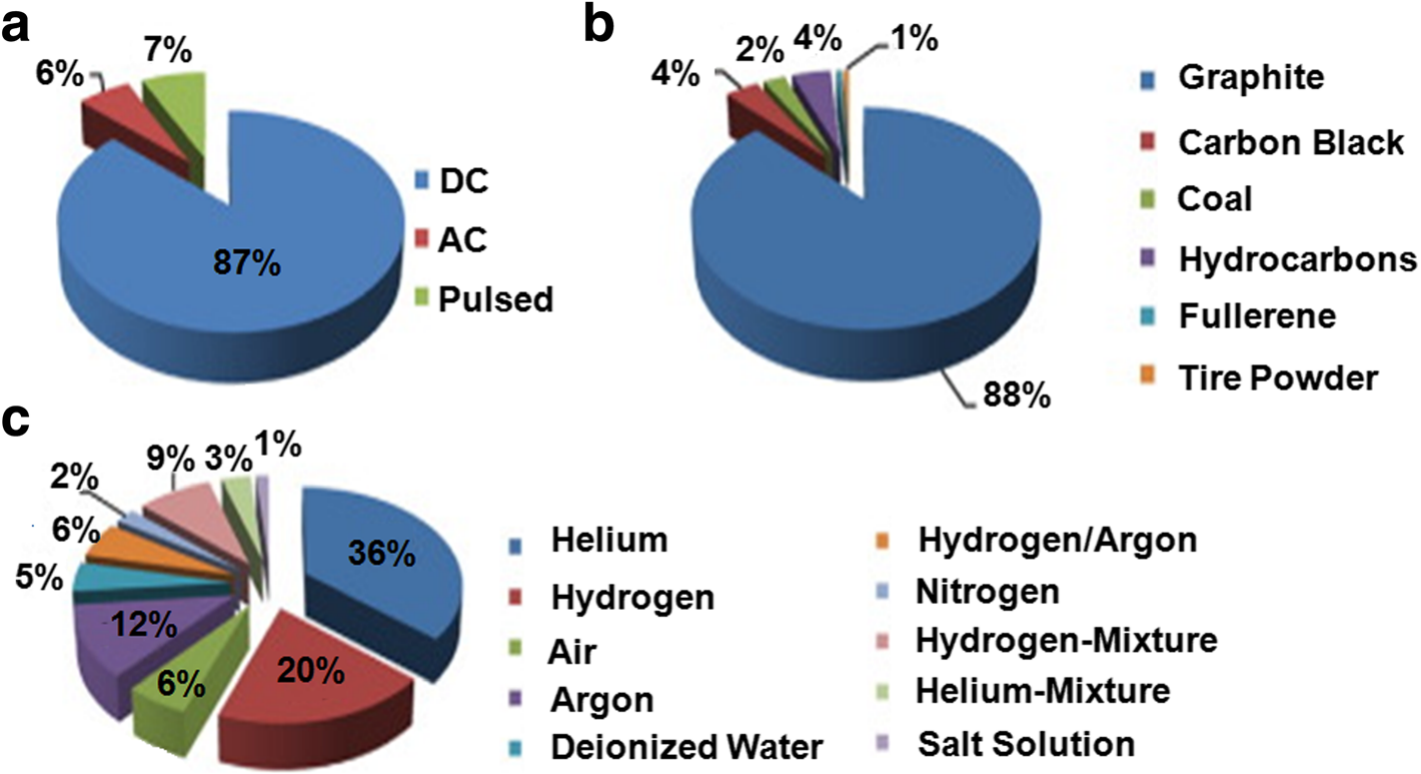
Pie chart showing percentage of published papers on AD-mediated CNT synthesis using **a** power supplies, **b** carbon precursors, and **c** atmospheres. The figure is modified and reprinted with permission from Elsevier [128]

Firstly, power supply is a must to create electron discharge, which ultimately controls plasma temperature. In order to generate arc between the electrodes, most of the researchers have used both AC and DC power supply. Figure 3a represents the popularity of AC, DC, and pulsed arc for CNT production. As shown in Fig. 3a, although DC power supply has been widely used, most of the time, the ionized gas in reaction chamber could drift towards cathode and thwarts the deposition of carbon ions on cathode surface. This can be overcome by using AC and/or pulsed AD. AC has shown to form the carbon deposits on the wall of chamber instead of cathode [125], and there is no established reason for this and has remained as an open area for future investigation. However, the major drawback of AC arc power is to get low yield of CNT. Our opinion is using pulsed arc, since they are more energetic and can be favorable for CNT production. This energetic pulse could bombard anode continuously that results in higher electron energy which could increase the yield [126].

Power supply also depends on voltage that is commonly applied between 15 and 30 V across the electrodes which should be kept constant for stable plasma. It has been shown that sudden changes in voltage can form bamboo-shaped CNT rather than straight one using AD method [127]. In addition to voltage, arc current has shown direct effect on the rate of CNT production. Most of the literatures have fixed non-fluctuated current of about 50–100 A in order to ablate the anode. But Cadek et al. [128] claimed that current density should be fixed in the range of 165–196 A/cm^2^ in order to increase CNT yield.

Secondly, selection of suitable carbon precursor in AD is very important for regulating the quality, especially purity of CNT. As shown in Fig. 3b, most of the published papers use graphite as carbon precursor might be because of its excellent heat and electron conductivity and also available in the market as pure form. Although some scientists have used carbon black because of its natural abundance, our opinion is to avoid this, since carbon black contains amorphous carbon which might add impurities to the final CNT product. Besides carbon black, coal has been found ideal for synthesizing FWCNT. Sulfur in coal has shown to accelerate the growth of FWCNTs and affects the diameter distribution of the CNT produced [129]. Albeit some other minor carbon sources such as fullerene [130], tire powder [131], and hydrocarbons [132] have been used, their contributions in CNT production need further understanding and investigations.

**Fig. 4:**
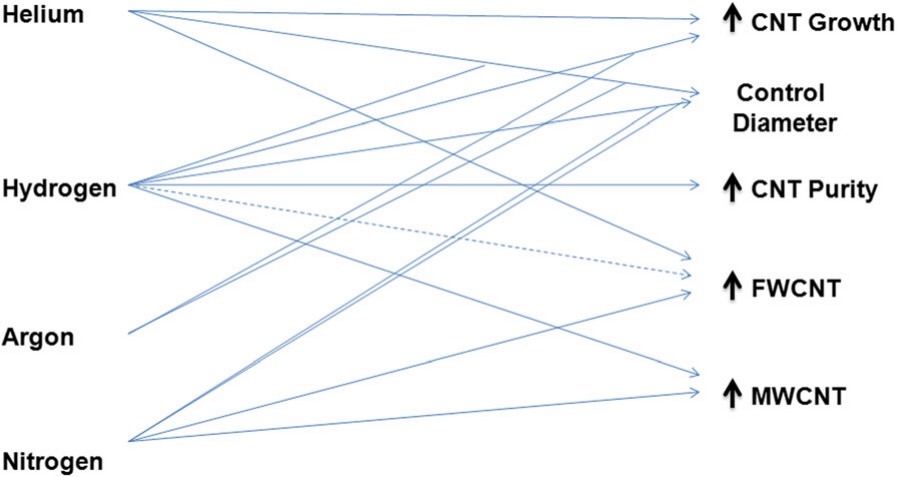
Effects of common gases used in CNT production in AD method. Straight and dotted lines indicate positive and negative effects, respectively. Intercepts of lines indicate synergistic effects

Thirdly, as shown in Fig. 3c, the highest percentage of studies conducted AD in presence of gases such as helium, hydrogen, and argon. Here, we summarize the effects of these three gases on CNT production and also show the effect of nitrogen on the rate of nanotube growth, diameter control, purity, and types of CNTs as shown in Fig. 4. Tang et al. [133] claimed that rapid launching of hydrogen could inhibit the ends of nanotube from closing. That is why, most of the scientists might use hydrogen in mixed with a noble gas like argon or helium to stabilize the plasma. Instead of adding gases, there are some liquids, e.g., deionized water and salt solution, which are used as shown in Fig. 3c. Some authors [134] used NaCl solution because of its good conductivity and cheaper than hydrogen, nitrogen, and helium gases, but its contribution to get high-quality CNT was not satisfactory.

**Fig. 5:**
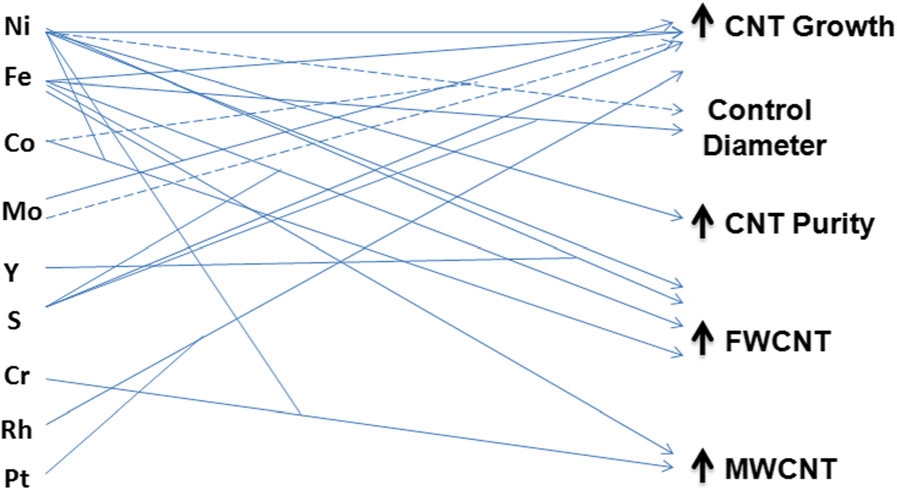
Effects of catalysts on CNT formation in AD method. Straight and dotted lines indicate positive and negative effects, respectively. Intercepts of lines indicate synergistic effects

Fourth, catalyst nanoparticle is a major parameter for CNT production. An ample literature study confirms us that most of the widely used catalysts are Ni, Fe, and Co. Sometimes, these catalysts have found to be used along with other catalysts as promoters. Here, we illustrate the effects of some common catalysts on CNT growth, diameter control, purity, and types as shown in Fig. 5. According to Table 1, there are a lot of bi(tri)metallic catalysts which are used for desired CNT production. But one should keep in mind that the size of CNT typically depends on appropriate size of metal catalyst particles. Most of the bi- or tri-metal catalysts might have long catalyst life span, but the size of this hybrid would be higher. However, Ni and Fe have remained as popular catalysts for the growth of high-quality CNTs. The major bottleneck of using Fe is their conversion to inactive catalyst form, e.g., Fe_2_O_3_. Although it is well known that MWCNT has been produced in absence of catalyst, a few reports have proved MWCNT formation in the presence of catalyst [135, 136]. Concentration of metal catalyst should be optimized first in order to optimize the nucleation rate. Wang et al. [137] claimed the maximum concentration of Fe in the anode must not exceed 10% for CNT formation. One hypothesis for this may be that higher amount of Fe could restrict the motion of carbon vapors towards cathode.

Fifth, pressure in arc chamber is important to release gas energy and maintain homeostatic environment, i.e., flow of ions between cathode and anode. Most of the published literatures used gas pressure between 300 and 700 Torr [138] which is the efficient pressure for hydrogen (500) [139], nitrogen (350) [140], argon (100) [141], helium (500) [142], air (60 Torr) [143]. Overall, it can be concluded that the optimum pressure can be fixed around 500 Torr for high yield of CNTs. However, experimentalists should perform design-of-experiment and optimize these pressure requirements based on other factors before settling optimum value.

At last, temperature is a major driven force in ionizing gas and creating plasma which is directly regulated with current density. It is believed that CNT synthesis at higher temperature could produce more crystalline CNTs. A higher temperature can be achieved (3600–3800 K) by using hydrogen plasma, whereas argon plasma needs in between 2200 and 2400 K [138]. Temperature requirement follows the trend of MWCNT > DWCNT > SWCNT as studied by Joshi et al. [144]. Although higher temperature could accelerate the CNT yield, it could have alternative effects on diameter control, e.g., reducing SWCNT diameter [145]. Zhao et al. [146] hypothesized that optimum temperature was 600 °C for CNT formation beyond which diameter of its decreases.

Overall, in order to improve CNT yield with desired quality, the above parameters should be overhauled seriously, since AD method cannot produce pure CNTs for large-scale uses. Producing high yield of CNT also depends on cooling rate, which also depends on arc current and cathode shape, temperature, and thermal conductivity of chamber gases. In general, 20–50 mg of CNT can be produced per minute of reaction which can be further boost up by optimizing current and pressure values, electrode diameters, catalyst composition, and atmosphere. Although some literatures have achieved to get a high yield of CNT 2–6 g/h [139, 147], controlling nanotube sizes and diameter is still unanswerable.

### CNT Growth in LA

**Fig. 6:**
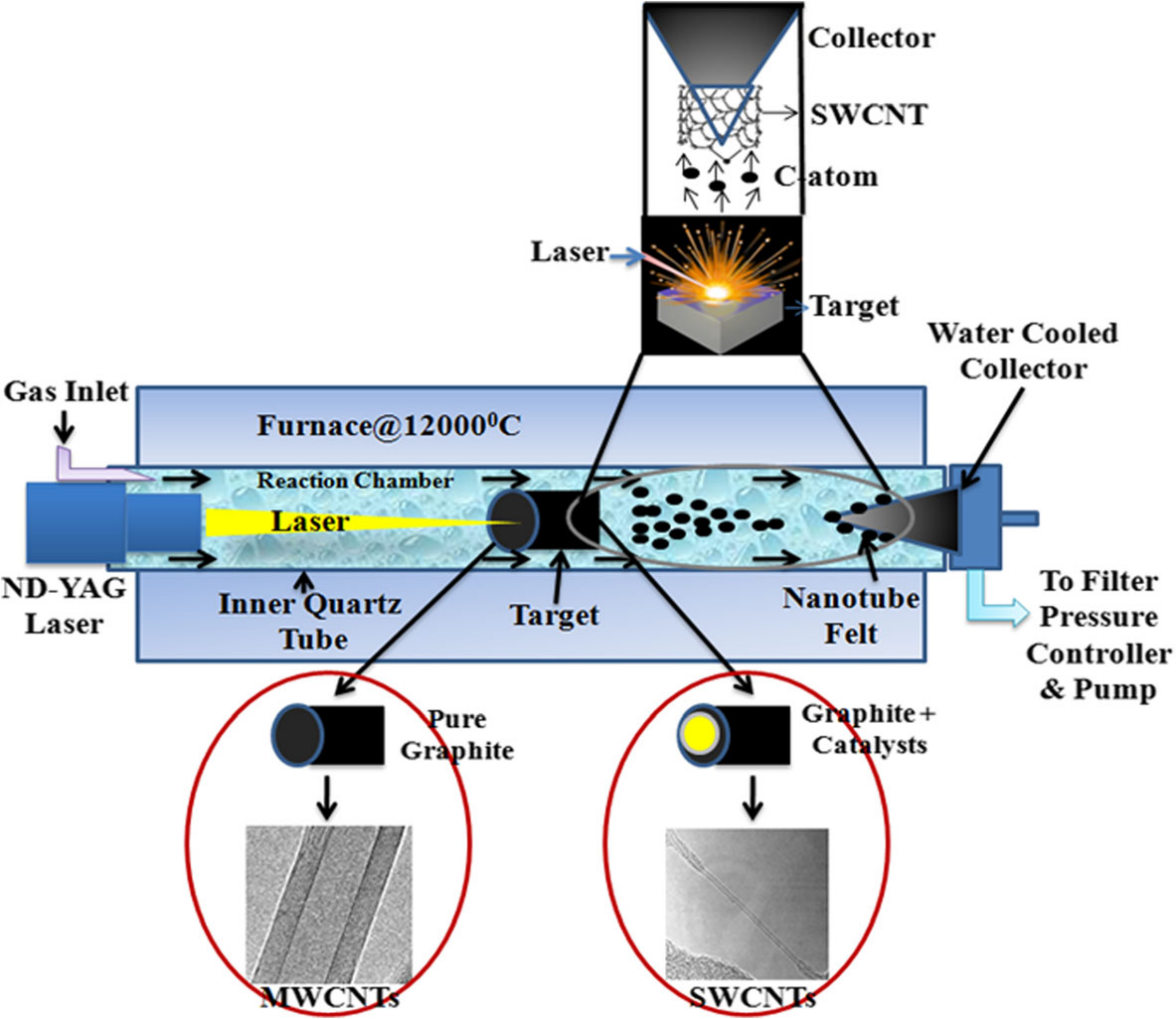
Schematic representation of a LA setup using ND:YAG laser system. MWCNT and SWCNT are synthesized when target rod is pure graphite and graphite catalyst mixture, respectively

Even though AD method has the scope of using suitable catalysts and gas phases, it has been remaining a longstanding problem to synthesize uniform and pure SWCNTs [88]. Therefore, the paradigm was shifted to invent another method called pulsed laser ablation process (PLAP) [148] or simply LA, capable of producing 500 mg of SWCNTs in 5 min with up to 90% purity [149]. The basic principle of LA is very simple and easy to perform. The specialty of this method is to use a light source, which is absent in AD process and was first introduced by Guo et al. [150]. The group proposed a model (Fig. 6) using pulsed ND:YAG (neodymium-doped yttrium aluminum garnet) laser source which is still now in use. The setup consists of a reaction chamber in a quartz tube (diameter 25 mm and length 1000–1500 mm) mounted in an adjustable hinged tube furnace/oven. Based on operator request, a target rod (either pure graphite for MWCNTs or metal graphite mixture for SWCNTs) is placed at middle high-temperature zone, usually operated at 1200 °C. The quartz tube is then sealed at downstream end to pump. An inert gas or mixed gas composite is then entered the quartz tube at the upstream side of the tube. The pressure controller at downstream side is controlled to feed gas flow into the tube. A laser source like ND:YAG is then entered the quartz tube and is placed in such a way that it directly focuses onto the target rod at the middle. The laser power evaporated the target rod and produced many scattered carbon species. The inert gas or its composite flows sweep the carbon species to deposit them in a collector at the downstream of the tube. Before the inert gas escaped from the tube, it enters a water-cooled collector and filter to deposit CNTs. Commonly, ND:YAG is operated under the following parameters (oscillation wavelength is either 1064 or 532 nm, heat is 300 mJ, repetition rate is 10 Hz, field width half maximum (FWHM) is <10 ns, and focused beam diameter on target spot is 3–8 mm).

#### Engineering Principles for CNT Yield Optimization

Table 2 displays the reaction parameters, and by engineering those, one can increase CNT yield using LA methods. CNT synthesis depends on many parameters such as the composition of target material, catalyst types and their specific ratio, gas flow [151], pressure, furnace temperature, and other laser properties (e.g., type, oscillation wavelength, heat, repetition, spot diameter, and others). Several hypotheses can be drawn by analyzing the events displayed in Table 2. Firstly, both SWCNT and MWCNT can be obtained when catalyst-doped graphite rod and pure graphite rod are used in LA method, respectively. Overall, LA can ensure higher yield of SWCNT with better properties and narrower size as compared with the SWCNT produced by AD method. Most of the obtained SWCNT are relatively pure than the SWCNT produced by AD. Secondly, most frequently used catalysts are Ni, Co, Fe, and Pt, and very often, there are promoters (i.e., mixed catalysts) for higher SWCNT yield. The efficiency of the catalyst follows the trend of Ni > Co > Pt or bimetallic Co/Ni > Co/Pt > Ni/Pt > Co/Cu. The highest yield can be ensured using Ni/Y and Ni/Co. Thirdly, argon is used more frequently in LA and was suitable for SWCNT production. Fourth, most of the ablating pressure falls in between 200 and 500 Torr. It envisions less than 200 Torr can produce amorphous carbons while higher temperature can produce crystalline CNT. Fifthly, higher temperature (>1000 °C) is favorable for CNT production with minimal defects. Temperature <1000 °C can induce defects and decrease CNT yield. Sixth, both the ND:YAG and continuous wave (cw)-CO_2_ laser ablations are used more frequently, but cw-CO_2_ laser ablation has shown better result than ND:YAG at lower temperature. Solar light energy could be economical, but target graphite evaporation may not be homogenous.

**Table 2:** Reaction parameters for CNT yield optimization using LA method

Target material	Metal catalyst (%)	Inert gas	Pressure (Torr)	Furnace/oven temp. (°C)	Laser properties (laser vaporization pulse)				Major observation	Refs
					Type	Oscillation wavelength (nm)	Heat (mJ/pulse)	Spot diameter (mm)		
Graphite rod	–	Ar	500	1200 900 200	Nd:YAG	532	250	3 and 6	• MWCNTs of length 300 nm are obtained with 4–24 layers • Yields depend on the following temp.: (a) At 1200 °C, defect-free MWCNTs are obtained; (b) At 900 °C, number of defects is increased; and (c) At 200 °C, no MWCNTs are synthesized • SWCNTs are not synthesized	[150]
Metal-graphite rod	Co (1) Cu (0.6) Nb (0.6) Ni (0.6) Pt (0.2) Co:Ni (0.6/0.6) Co:Pt (0.6/0.2) Co: Cu (0.6/0.5) Ni/Pt (0.6/0.2)	Ar	500	1200	Nd:YAG	532	300	6–7	• SWCNT are obtained with increasing temp up to 1200 °C • Yields obtained in an order of (a) Ni > Co > Pt, (b) Co/Ni > Co/Pt > Ni/Pt > Co/Cu • For Cu and Nb: no SWCNTs are secured	[48]
Metal-graphite rod	Ni-Co (1.2)	Ar	500	1200	Nd:YAG	532 (initial)	250 (initial)	5 (initial)	• >70% SWCNTs are secured with uniform diameter in the form of rope • One rope contains from 100 to 500 SWCNTs	[49]
						1064 (final)	300 (final)	7 (final)		
Metal-graphite rod	Co/Ni (1)	Ar	500	200	Nd:YAG	532	490	6	• When the flow tube is 2.5 cm in diameter, Web-like SWCNT deposit is retained ↓ • When the tube dimension is increased by ~5 cm in diameter, 50 vol% of SWNCT is obtained ↓ • When 2.5 cm in diameter quartz tube coaxial and 5 cm tube extending are installed, 60 to 90 vol% SWCNT is acquired • Finally, 1 g/day SWCNTs are accomplished	[160]
Metal-graphite rod	Ni (2) Co (2) Fe (2) Y (0.5) Ni/Y (4.2/1, 2/0.5, 1/0.25, 0.6/0.6, 0.5/0.1) Ni/Co (4.2/1, 2/2, 2/0.5, 1/0.25, 0.6/0.6, 0.5/0.13, 0.4/0.4) Ni/Fe (4.2/1, 2/0.5, 0.6/0.6)	Ar	400	No Furnace is used	CO_2_ 250 W	–	–	–	• The soot contains large amounts of clean bundles of SWCNT (diameter 20 nm and lengths >1 μm) • SWCNTs of 80 vol% are synthesized at a rate of 50 mg/h • Ni/Y or Ni/Co showed rubbery web-like SWCNT soot	[50]
Graphite + catalysts powder	Co Ni Y (2)	Ar	187–337	–	Solar energy (2000 W)	–	–	–	• Parallel and bundled SWCNTs are obtained	[156]
Metal-graphite rod	Ni (2) Co (2) Y (0.5) Fe (2) Ni*/*Co (4.2*/*1, 2/ 2, 0.5/ 1, 0.6/0.6, 0.5*/*0.1) Ni*/*Y (4.2*/*1, 2/0.5, 1*/*0.25, 0.6*/*0.6, 0.5*/*0.1) Ni*/*Fe (4.2*/*1, 2/0.5, 0.6*/*0.6) Co*/*Y (2/0.5) Ni/La (2*/*0.5)	Ar N_2_ He	400	–	CO_2_ 12000 W	–	–	0.16	• SWCNTs of diameter 1.4 nm are self-organized into a 20 nm bundle • Maximum yield was noticed with Ni*/*Y and Ni*/*Co catalysts • Cw-CO_2_ laser ablation is an easy and environment favorable method for the growth of high-quality SWCNT	[158]
Metal-graphite rod	Co and Ni (2.5)	Ar N_2_	750	1100 °C	CO_2_ 2100 W	–		–	• 20–40% SWCNT with mean diameter of 1.2–1.3 nm are obtained	[190]
Metal-graphite rod	Co–Ni (0.6)	Ar	500	25–1150	UV	248	–	–	• SWCNTs with 15–20 nm in diameter are secured • Yield is lower at lower temp • Repetition rate of 30 to 150 Hz leads to a higher yield and larger bundles	[157]
Metal-graphite rod	Ni (1) Co (1)	Ar N_2_	600	7726	ND:YAG	532	240	–	• N_2_ atmosphere produces more bundled than those of Ar ambience	[191]
Metal-graphite rod	Co Ni (1.2)	Ar	750	999 1099 1199 1349	UV	308	58	–	• SWCNTs are formed with diameter 1.2∼1.7 nm and length >2 μm • The highest yields at 1349 °C	[192]
Metal-graphite rod	Co Ni	Ar	500	1100	UV	248	–	–	• SWCNT deposit of diameter 1.2 nm is secured	[193]
Metal-graphite rod	Co/Ni	Ar	500	900 1000 1150	UV	248	–	–	• SWCNT of diameter 1.2 nm and length 10 μm aggregate into bundles containing 2–40 nanotubes • Optimal catalyst concentration of 1.2% synthesis high-quality SWCNT • Increasing the furnace temperature increases diameters	[194]
Graphite rod	Fe_2_O_3_ (1–5)	Ar	500	–	ND:YAG	532	–	–	• Web-like MWCNT structures are obtained • Fe_2_O_3_ catalyst (1%) influenced the magnetic properties of the CNTs	[195]
Graphite rod	–	Ar	50 150 400 760	–	CO_2_ 3.5 kW	–	–	2.6	• Diameter of obtained MWCNT is in the range of 5–40 nm • Pressure at 760 Torr was more effective to get large fraction MWCNT	[196]
Graphite rod	Fe/Al, Co/Al Ni/Al (1:1)	N_2_ H_2_	–	800	–	–	–	–	• Synthesized different nanostructures • Fe in the catalyst mix yielded only MWCNT • Samples containing Co or Ni led to a mixture of MWCNT and SWCNT	[197]

**Fig. 7:**
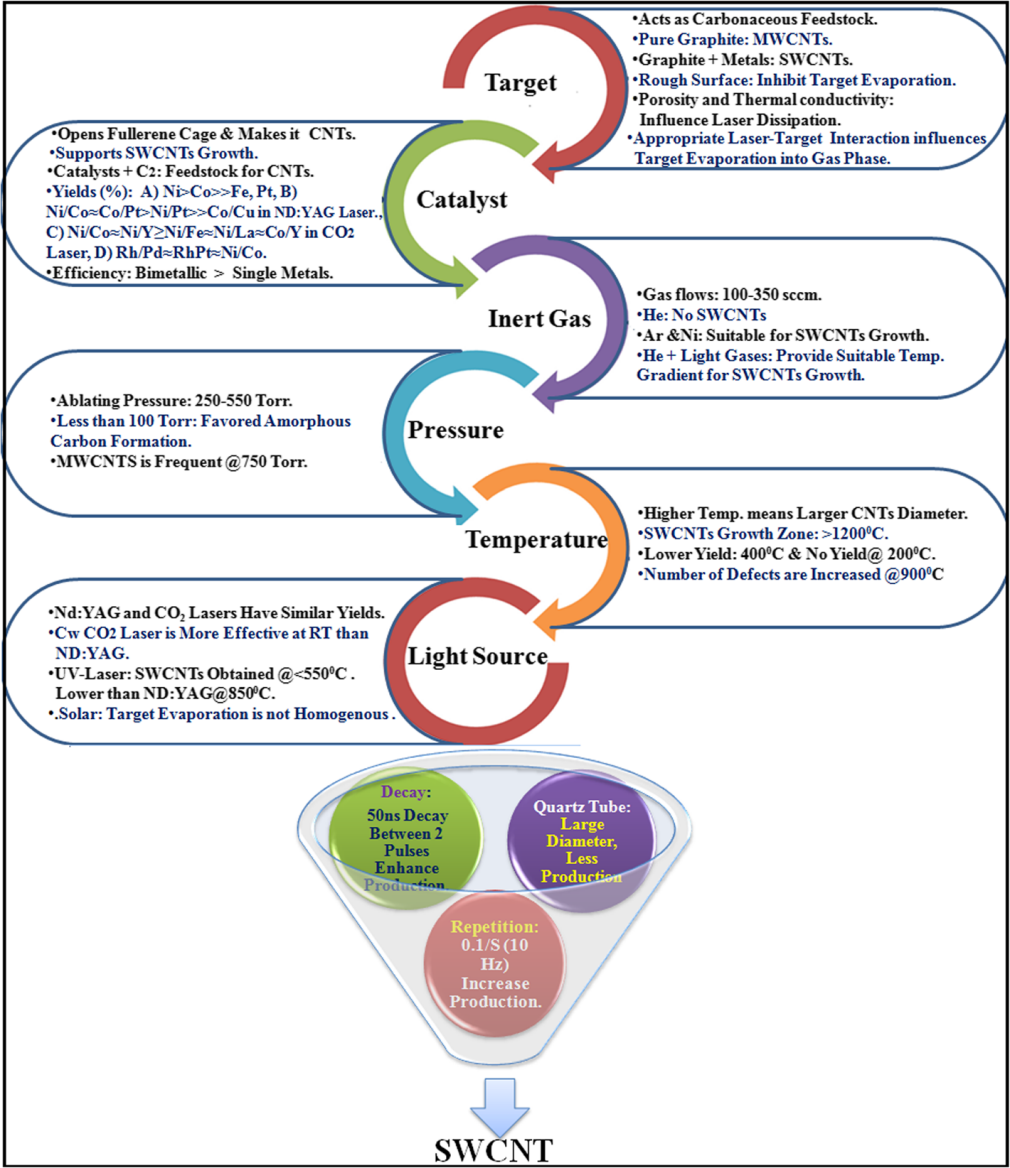
Rate-limiting steps and major factors regulating the growth of SWCNT

Various rate-limiting steps and some essential factors such as target material, catalysts, gas types, pressure, temperature, and light sources regulate CNT production in LA process. Their interactions along with key factors are depicted as nutshell in Fig. 7. Not only target graphite rod but also carbon atoms suspended in surrounding atmosphere in reaction chamber can act as carbon feedstock for nanotube growth [152]. Available supply of carbon feedstock allows the tuning of CNT growth in reaction zone to ensure the formation of pure and defect-free CNTs. Fullerene is formed as an intermediate product in reaction zone during CNT synthesis [152]. The intermediate fullerenes may be degraded into lower carbon fragments (C_2_, C_3_, etc.) by laser effects, and the disintegrated carbon fragments may act as feedstock for further CNT growth.

Figure 7 reveals some of the catalysts studied for the synthesis of SWCNTs by laser technique. Each metal catalyst poses specific catalytic growth effect and synthesizes SWCNTs with a great variation. The major role of a given catalyst is to open cage structure of fullerenes in a reaction chamber followed by CNT initial cluster formation. The cluster then acts as base from where microtubule-like nanotube grows. It runs until the catalyst cluster becomes too large to inhibit carbon evaporation and diffusion through or over the catalyst surface [49, 150]. The deposited carbon layer might be saturated to accept further small carbon fragments and stop nanotube growth. Pulsed laser heats target graphite metal rod that also produces many molten carbon and catalyst mixture in gas phase [52, 53]. These ejected molten carbon-catalyst clusters are then cooled followed by disintegration. The segregated carbons are then deposited onto collector, and SWCNT growth occurs.

However, the actual roles of a given catalysts in nanotube growth is not well reported. Further study is necessary to understand the role of a single or a combination of certain catalysts necessary for CNT growth. It has been documented that a bimetallic mixture is more efficacious than a single one [150]. Most frequent metal catalysts used in ND:YAG, CO_2_, and solar lasers are Ni/Co (0.6/0.6 at.%) and Ni/Yt (4/1 and 2/0.5 at.%) [149]. The catalyst mixture must be used at low concentration since high catalyst concentration contaminates the final product and exists as impurities in SWCNT. The purification of contaminated SWCNTs is challenging but necessary for many industrial applications especially for optoelectronics and also for reducing environmental impact.

Gas flow and its pressure are considered as the most prominent rate-limiting factors in LA process. Zhang et al. [51] studied the synthesis of SWCNTs in both N_2_ and Ar gas under various conditions. They observed CNT growth at higher temperature where N_2_ is eliminated by the ablation products. Both Ar and N_2_ demonstrated similar effects, whereas He showed no effects on CNT formation. The inert gas atmosphere probably speeds up wave expansion and thermal conductivity in CNT growth process. At lower pressure (about 100 Torr), amorphous carbon formation is favored over SWCNT formation. SWCNTs begin to form at/above 200 Torr. The rate of CNT formation strongly enhances at/above 600 Torr [153]. The pressure effect depends on the metal concentrations and laser types used in reactions. Low-pressure threshold could be 100 Torr at appropriate metal concentration and accurate LA [153].

Since SWCNTs have to be collected from water-cooled chamber of LA process, the formation of SWCNTs occurs at/near graphite target in the proximity of laser attack surface. Appropriate temperature has to be maintained in reaction chamber to get greater target ablation by laser irradiation. The hot plasma plume is generated due to high-temperature ablation of target material which supports proper and fast SWCNT growth in gas phase [88, 154]. Kokai et al. [53] investigated nanotube growth rate under normal oven temperature. SWCNTs having larger diameter were produced at higher temperatures than those of lower temperature. Takizawa et al. [54] obtained similar temperature effects on CNT formation. The continuous supply of hot carbon clusters might stimulate and activate the active sites of other carbon fragments that are supposed to clump together during nanotube synthesis. The optimum growth zone for SWCNT formation should maintain >1200 °C. Lower temperature creates nanotuber of defects and stimulates the formation of amorphous carbon. The actual mechanism of temperature in reaction chamber is to facilitate the vaporization of target feedstock as well as support the assembly processes of smaller carbon fragments like C_2_ and C_3_ in CNT growth in gas phase.

**Fig. 8:**
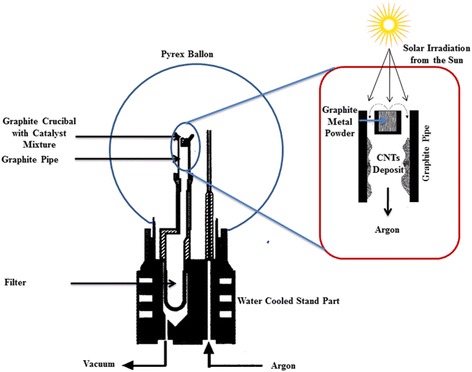
Schematic representation of experimental setup for a solar evaporation system. Target and pipe are heated by the incident solar radiation from the Sun. The hot channel between the target and pipe acts as local furnace, avoiding the need of external furnace. The figure is adapted with permission from Elsevier [156]

The target evaporation is realized by different lasers at different wavelengths [155] or light sources such as ND:YAG, UV lasers, cw-CO_2_, and solar energy [50, 150, 156, 157]. LA of target material can be accomplished using single- or double-pulsed lasers at various wave lengths rather than cw-CO_2_ laser beam [50, 153]. Although the two methods need almost the same apparatus and conditions, the main difference between them is the requirement of higher light intensity, i.e., 100 kW/cm^2^. The cw-CO_2_ laser does not require any external furnace in contrast to pulsed ND:YAG laser system. However, no big differences are noticed in terms of SWCNT growth in LA process. The cw-CO_2_ laser system is almost similar to arc ablation in terms of background gas and metal catalyst mixtures [152]. It is more effective for room temperature SWCNT synthesis using ND:YAG-pulsed laser system that requires high temperature [158]. Braidy et al. [157] used UV laser for the first time to vaporize target material at lower wavelength. The group has successfully synthesized SWCNT diameter of 15 to 20 nm at 550 °C where ND:YAG laser system needs 850 °C. Though ND:YAG, cw-CO_2_, and UV laser systems are effective, they need laser and large amount of power and thus are not economically favorable. Laplaze et al. [156] synthesized CNT using solar energy as an economic light source as shown in Fig. 8. Solar energy was captured in a Pyrex balloon flask, which was installed on top of a water-cooled support. The balloon acted as reaction chamber, trapped, and concentrated incident solar energy to maintain high temperature which was necessary for evaporating the target material. The material was then placed into a graphite crucible which consists of graphite and catalysts such as Ni/Co and Ni/Yt mixtures. The crucible was then mounted into a graphite pipe. The pipe was then connected to a water-cooled brass barrel. At downstream, an inert gas (Ar) was introduced through the graphite pipe. Another pipe was fitted to maintain the pump for regulating the gas circulation and pressure. A parabolic shape mirror was fixed on the target to capture sunlight. Using the setup, it was possible to collect and concentrate temperature of about 3000 K and could be reached at 2 kW when the sky is clean and fresh. This evaporated the target material to produce smaller carbon fragments which were then drawn through the graphite pipe and deposited onto the tube as nanotube soot material. The soot material may consist of MWCNT or FWCNT depending on the pressure applied and gas flow. Laplaze et al. [156] synthesized SWCNT of diameter 1.2–1.5 nm at a yield rate of 100 mg/h. The disadvantages of the method are the need of good weather, clean, and fresh sky to allow temperature optimum for CNT growth. In addition, the tubes were found contaminated with amorphous carbon and metal impurities, making a mandatory need of CNT purification prior to applications. Although the procedure is economical, the productivity is lesser than pulsed laser (ND:YAG) but comparable to CO_2_ laser approach. Both techniques need continuous wave power and very little setup procedure, since they do not need external furnace for high temperature [149].

In addition to major CNT growth factors in LA process, such as catalysts, temperature, laser source, and gas nature, other parameters such as pulse repetition, time decay between two pulses [159], and inner quartz tube size have to be tuned to get optimum yield. Repetition of laser pulses is necessary to localize suspended carbon cluster in the reaction zone. Thess et al. [49] repeated the incident of ND:YAG pulses (the first one is at 532 nm and second one is 1064 nm) with delay of about 50 ns. They achieved higher SWCNT yield of >70%. Yudasaka et al. [52] found better result at intervals of 0.1/s. However, no significant change was observed when the decay was between 0.1 and 120 s. The delay between two pulses ionizes the plume expands sufficiently [152]. Between the times of the pulses, the target material surface is reorganized to be cooled significantly.

Thess et al. [49] designed inner quartz tube with 25 mm in diameter inside a 56-mm outer tube. They passed argon gas through inner tube and observed that the tube size helps to confine LA plume and supports appropriate gas flow to form CNTs. They hypothesized that an increase in inner tube diameter could decrease the rate of CNT formation. The placement of inner reaction tube in front of the target plays vital role in nanotube formation. Rinzler et al. [160] explained the use of a typical 25-mm quartz tube. It increased the possibility of getting 50 vol% SWCNTs. To optimize yields, they extended 25-mm quartz tube to a 50 mm one. This leads to increase SWCNT yield by 90 vol%. Probably, the actual role of appropriate tube size is to maintain homogenous gas and straight laser flow towards the target material to generate plume rapidly without lifted off and simultaneously decreases target pitting. In addition, plugging of an appropriate narrow ~2.5 cm rather than bigger tube will support to concentrate the laser light to fall on target surface to maintain appropriate temperature for target ablation. It can reassemble the free catalyst atoms suspended in gas phase and confine them to control target dissipation. Therefore, through target re-vaporization, it increases and provides new carbon feedstock for nanotube growth. We believe the further scaling-up of the original design would provide better yield even in few seconds to minutes.

Most of the authors adjusted both major and minor parameters to adjust optimal conditions to overcome the problem they faced during ablation process of CNT formation. We should not avoid any single parameter to get maximum yield since they are part of a cascade of regulated interactions. Fine tuning of all parameters especially the temperature and laser type is necessary for the rapid conversion of vaporized small carbon fragments to nanotubes. To the best of our knowledge, only few types of laser sources are available and they are not economically favorable and environment-friendly except solar energy. Findings of alternative target material rather than pure graphite as major carbon sources such as coal, charcoal, and asphalt would give prominent way to reduce the cost of SWCNT synthesis, since it would reduce the starting material costs by approximately tenfold.

### Chiral Controlled CNT Synthesis Using AD and LA

The CNT chirality, especially SWCNT along with its diameter, determines its electrical properties with the chiral numbers [161]. In general, when *n* = *m*, they are “armchair” SWCNTs, being metallic with a zero bandgap; when *n* − *m* = 3*i* and *i* ≠ 0, the SWCNTs have a very small bandgap and are generally considered as metallic; and when *n* − *m* = 3*i* ± 1, the SWCNTs are semiconducting. Lastly, the SWCNTs with chiral indices of (*n*, 0) are referred to as “zig-zag,” and they could be either metallic or semiconducting [162]. Nearly 67% of SWCNTs are semiconducting, and 33% of them are metallic among all possible (*n*, *m*) combinations [163]. This indicates that the CNTs that we obtained from AD, LA, and CVD methods are a mixture of nanostructures with a broad distribution in diameter and chirality. As a corollary, isolating identical SWCNT structures (i.e., same diameter and the same chirality) has remained as a major challenge for large-scale nanotube uses.

**Fig. 9:**
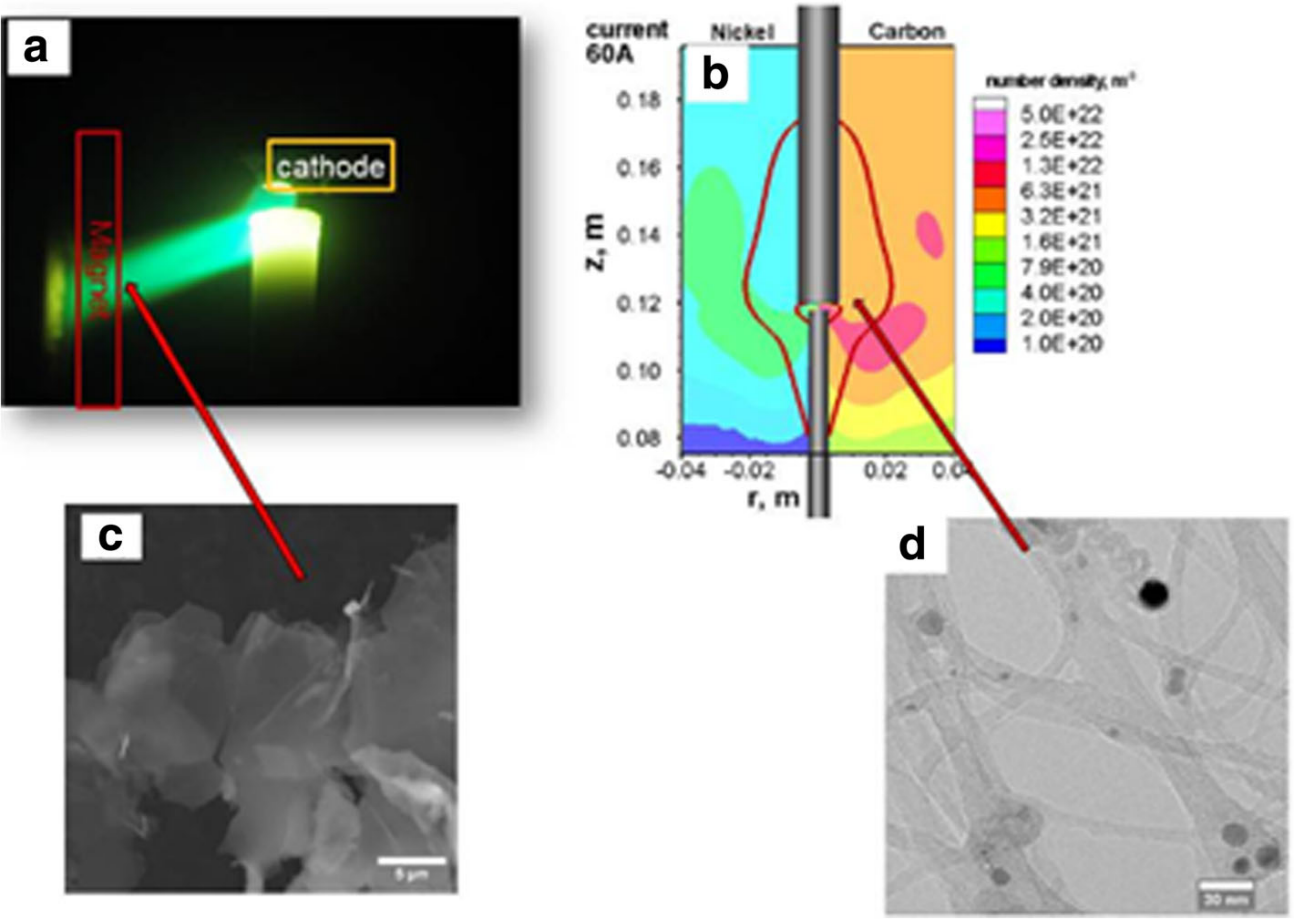
Photo of the applied magnetic field at arc plasma (**a**), computed carbon and catalyst particle density distribution showing the regions with preferable conditions for chiral CNT synthesis (**b**), scanning electron microscope image of graphene sheets (**c**), and TEM image of SWCNT bundles with specific chirality (**d**). The figures are adapted with permission from Institute of Physics Science [166]

In order to ensure chiral CNT structures, two approaches have been intensively examined: firstly, the post-synthesis separation according to the chiral vectors (*n*, *m*) of CNT relies on affinity approach, surfactant pattern approach, etc. which have been reviewed by Zhang and Zeng [164], and secondly, it is also possible to control CNT chirality during direct synthesis of CNTs with the same (*n*, *m*). Although extensive efforts towards the controllable growth of CNTs with specific diameter, chirality, and conducting type have been made only by using CVD, there is only few reports published dealing with AD and LA for examining and defining chiral CNT synthesis. By controlling the temperature in the range of 780 < T < 1050 °C in LA method, Bandow et al. [165] tried to tune the diameter of the tubules from 0.81 to 1.51 nm. Most of the chiral vectors (*C*) relevant to this work were assigned to the armchair tube direction (*n*, *n*), and the threshold temperature for significant SWCNT production was found to be 850 °C. Recalling one of the main drawbacks of AD method is relatively little control over the alignment (i.e., chirality) of the created CNT. Thence, Keidar et al. [166] used magnetic fields to control SWCNT diameter and chirality as shown in Fig. 9. The authors claimed that the obtained SWCNTs formed by rolling the lattice of graphene at different angles which might create a visible twist, chirality, or spiral in the SWCNT molecular structure, though the overall shape remains cylindrical. These results suggest that the length, diameter, and thus chirality of arc-produced SWCNTs can be controlled by an external magnetic field applied to the discharge. A magnetic field of a relatively small magnitude of several kilograms was found to result in a dramatically increased production of smaller diameter (about 1 nm) of SWCNTs and a broadened spectrum of diameters/chiralities of synthesized SWCNTs. A particular conclusion from this work is that the basis of the control of the AD synthesis lies in the fundamentals of the catalyst formation and interaction of the catalyst with the active carbon species. It was also found that, dependent on the magnetic field configuration used, few-layer graphene sheets can be formed. In fact, this led to a single-step simultaneous synthesis and magnetic separation of high-quality graphene flakes and SWCNTs in magnetically enhanced AD plasmas. Based on these results, one can suggest an active electromagnetic device that might be introduced for the control of the catalyst nanoparticles’ nucleation and the SWCNT and graphene growth. Another strategy has recently been published by Fang et al. [121] who synthesized semiconducting SWCNT with diameter of about 1.5 nm by using an actuator-driven high-speed system that is able to extract material from the arc plasma volume during the synthesis procedure.

## Conclusions

We critically review the advancement of AD and LA for controlled SWCNT and MWCNT synthesis. Although many methods are available, it is not surprising that novel methods or ways are coming out everyday due to the inability of a single method to yield high-quality CNT for industrial uses. We highlight the novel growth and nucleation mechanisms of CNTs with simplified illustrations, which are easily understandable to ordinary experimentalists, potential readers in the fields, and also non-specialists. Although the basic mechanism behind them is rather simple, the ingredients such as carbon feedstocks, catalysts, substrates, and temperature effects have found as nerve centers for yielding industrial grade CNTs in megaton volumes. By tuning these parameters, it is possible to control the overall reaction conditions and would help to get SWCNT and MWCNT with discrete properties, quality, crystallinity, chirality, higher yield, and architectures. Overall, the production rate of AD can ensure higher yield of CNT than that of LA. Although both methods are popular for synthesizing SWCNT, most of the SWCNTs produced by AD are shorter as compared with long bundles of SWCNTs produced by LA. Synthesizing MWCNTs using AD and LA is not very much interesting as both methods are expensive. Both AD and LA methods can produce CNT with few structural defects, but the reaction product from LA is quite pure than that from AD method. Some investigations should be carried out in order to further improve the AD and LA methods, so that higher quality and quantity of CNTs can be guaranteed. Literatures published on the investigations of most significant parameters of AD and LA have remained controversial, and further experimentations should be conducted to understand the vital roles of carbon precursors, temperatures, pressure, catalyst type and shapes, and geometry of electrodes/targets. Minimizing CNT synthesis cost has remained a big challenge. Most of the authors have been used expensive graphite as a precursor, whereas other cheapest carbon materials like carbon black and coal have not been utilized extensively and are a potential area of investigation. Scientist can also look after to use naturally abundant carbon sources, e.g., activated carbon and plant-based materials. In order to maximize the AD and/or LA production rate, we suggest using mathematical or theoretical models before optimizing the experimental reaction parameters. It will help to correlate between the predicted value and experimental findings which will further improve understanding of the CNT growth mechanisms involved in both processes. They may also have resolved problems hindering the effectiveness of the processes.

## Additional file

Additional file 1: Figure S1. Schematic representation of the birth of CNT through various routes. (DOCX 2313 kb)

## Abbreviations

AD: Arc discharge
CNT: Carbon nanotube
CVD: Chemical vapor deposition
DWCNT: Double-walled carbon nanotube
FECA: Foreign element catalytic agent
FWCNT: Few-walled carbon nanotube
HET: High-energy site
LA: Laser ablation
MWCNT: Multi-walled carbon nanotube
PLAP: Pulsed laser ablation process
SDL: Screw-dislocation-like
SLS: Solid–liquid–solid
SWCNT: Single-walled carbon nanotube
TWCNT: Triple-walled carbon nanotube
VACNT: Vertically aligned carbon nanotube
VLS: Vapor–liquid–solid
VSS: Vapor–solid–solid
