# Supplementary material for: Can We Optimize Arc Discharge and Laser Ablation for Well-Controlled Carbon Nanotube Synthesis?
Source: Nanoscale Res Lett. 2016 Nov 18;11:510. doi: 10.1186/s11671-016-1730-0 (PMC5116021; doi:10.1186/s11671-016-1730-0)
Supplement: Additional file 1: Figure S1. — Schematic representation of the birth of CNT through various routes. (DOCX 2313 kb) [file 11671_2016_1730_MOESM1_ESM.docx]

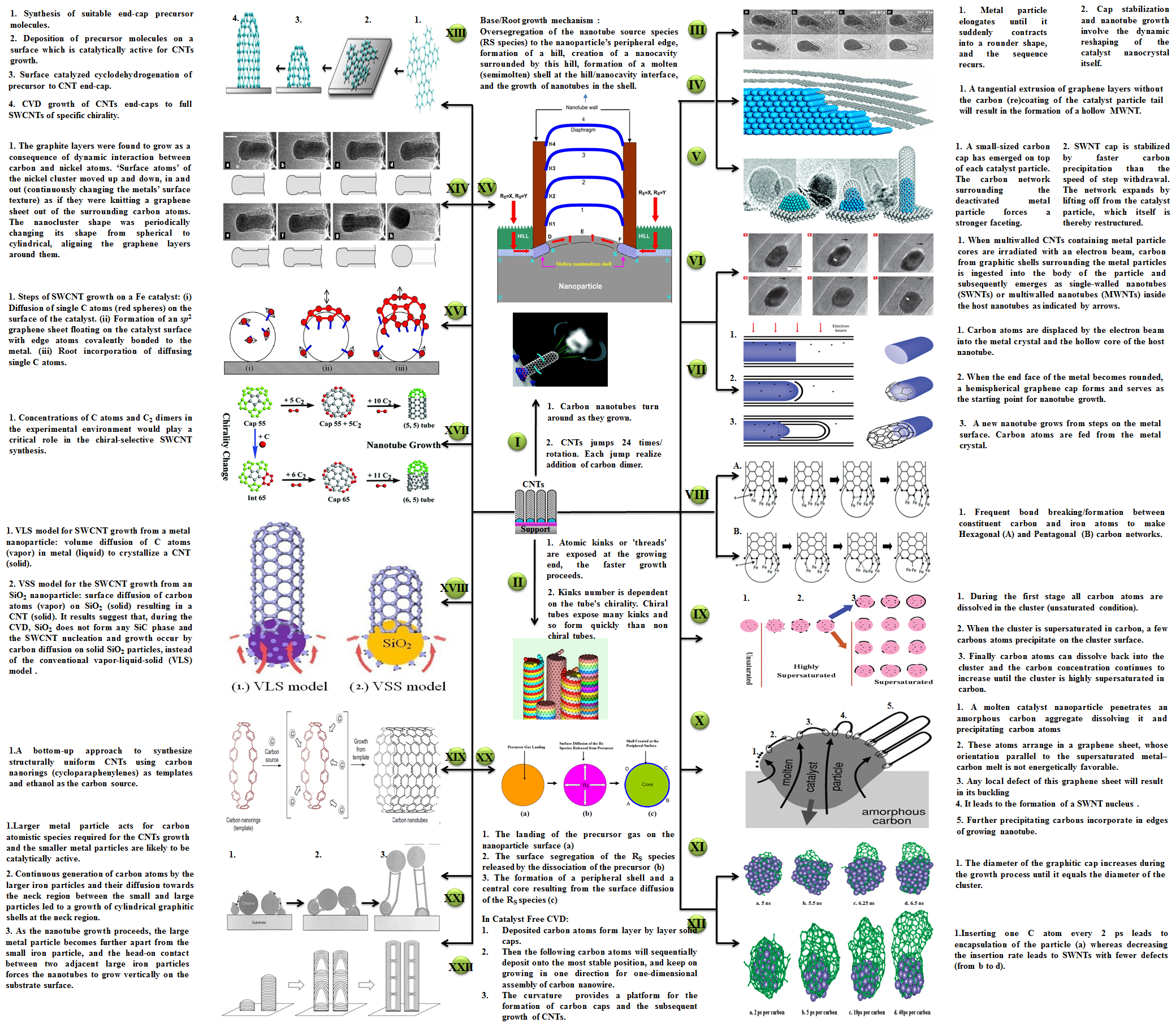


**Additional file 1: Fig. S1** Schematic representation of the birth of CNT through various routes. Figures are redrawn and edited with permissions from Royal Society of Chemistry; American Chemical Society; Nature; Elsevier and American Physical Society [1-14].

**References**

[1] Marchand M, Journet C, Guillot D, Benoit J-M, Yakobson BI, Purcell ST. Growing a carbon nanotube atom by atom:“and yet it does turn”. Nano Lett. 2009;9(8):2961-6.

[2] Ding F, Harutyunyan AR, Yakobson BI. Dislocation theory of chirality-controlled nanotube growth. Proc. Natl. Acad. Sci. U.S.A. 2009;106(8):2506-9.

[3] Chen Y, Zhang J. Diameter controlled growth of single-walled carbon nanotubes from SiO_2_ nanoparticles. Carbon. 2011;49(10):3316-24.

[4] Omachi H, Nakayama T, Takahashi E, Segawa Y, Itami K. Initiation of carbon nanotube growth by well-defined carbon nanorings. Nature Chem. 2013;5(7):572-6.

[5] Li D-C, Dai L, Huang S, Mau AW, Wang ZL. Structure and growth of aligned carbon nanotube films by pyrolysis. Chem Phys Lett. 2000;316(5):349-55.

[6] Hofmann S, Sharma R, Ducati C, Du G, Mattevi C, Cepek C, et al. In situ observations of catalyst dynamics during surface-bound carbon nanotube nucleation. Nano Lett. 2007;7(3):602-8.

[7] Rodriguez-Manzo JA, Terrones M, Terrones H, Kroto HW, Sun LT, Banhart F. In situ nucleation of carbon nanotubes by the injection of carbon atoms into metal particles. Nature Nanotechnol. 2007;2(5):307-11.

[8] Page AJ, Ohta Y, Irle S, Morokuma K. Mechanisms of Single-Walled Carbon Nanotube Nucleation, Growth, and Healing Determined Using QM/MD Methods. Acc Chem Res. 2010;43(10):1375-85.

[9] Ding F, Bolton K, Rosen A. Nucleation and growth of single-walled carbon nanotubes: A molecular dynamics study. J Phys Chem B. 2004;108(45):17369-77.

[10] Mueller A, Amsharov KY, Jansen M. Synthesis of end-cap precursor molecules for (6,6) armchair and (9,0) zig-zag single-walled carbon nanotubes. Tetrahedron Lett. 2010;51(24):3221-5.

[11] Helveg S, Lopez-Cartes C, Sehested J, Hansen PL, Clausen BS, Rostrup-Nielsen JR, et al. Atomic-scale imaging of carbon nanofibre growth. Nature. 2004;427(6973):426-9.

[12] Mohammad SN. Systematic investigation of the growth mechanisms for conventional, doped and bamboo-shaped nanotubes. Carbon. 2014;75:133-48.

[13] Raty J-Y, Gygi F, Galli G. Growth of carbon nanotubes on metal nanoparticles: a microscopic mechanism from ab initio molecular dynamics simulations. Phys Rev Lett. 2005;95(9):096103.

[14] Wang Q, Ng MF, Yang SW, Yang Y, Chen Y. The mechanism of single-walled carbon nanotube growth and chirality selection induced by carbon atom and dimer addition. ACS Nano. 2010;4(2):939-46.
